# Development of a clinical scoring system for assessment of immunosuppression in patients with tuberculosis and HIV infection without access to CD4 cell testing – results from a cross-sectional study in Ethiopia

**DOI:** 10.3402/gha.v7.23105

**Published:** 2014-02-13

**Authors:** Sten Skogmar, Taye T. Balcha, Zelalem H. Jemal, Jonas Björk, Wakgari Deressa, Thomas Schön, Per Björkman

**Affiliations:** 1 Infectious Diseases Research Unit, Department of Clinical Sciences in Malmö, Faculty of Medicine, Lund University, Sweden; 2 Health Ministry, Addis Ababa, Ethiopia; 3 Oromia Regional Health Bureau (ORHB), Addis Ababa, Ethiopia; 4 Research and Development Unit, Skåne University Hospital, Lund, Sweden; 5 Department of Public Health, Addis Ababa University, Addis Ababa, Ethiopia; 6 Department of Medical Microbiology, Faculty of Health Sciences, Linköping University, Sweden; 7 Department of Clinical Microbiology and Infectious Diseases, Kalmar County Hospital, Sweden

**Keywords:** HIV, tuberculosis, Ethiopia, scoring system, CD4 cell, timing of ART

## Abstract

**Background:**

Currently, antiretroviral therapy (ART) is recommended for all HIV-positive patients with tuberculosis (TB). The timing of ART during the course of anti-TB treatment is based on CD4 cell counts. Access to CD4 cell testing is not universally available; this constitutes an obstacle for the provision of ART in low-income countries.

**Objective:**

To determine clinical variables associated with HIV co-infection in TB patients and to identify correlations between clinical variables and CD4 cell strata in HIV/TB co-infected subjects, with the aim of developing a clinical scoring system for the assessment of immunosuppression.

**Design:**

Cross-sectional study of adults with TB (with and without HIV co-infection) recruited in Ethiopian outpatient clinics. Clinical variables potentially associated with immunosuppression were recorded using a structured questionnaire, and they were correlated to CD4 cell strata used to determine timing of ART initiation. Variables found to be significant in multivariate analysis were used to construct a scoring system.

**Results:**

Among 1,116 participants, the following findings were significantly more frequent in 307 HIV-positive patients compared to 809 HIV-negative subjects: diarrhea, odynophagia, conjunctival pallor, herpes zoster, oral candidiasis, skin rash, and mid-upper arm circumference (MUAC) <20 cm. Among HIV-positive patients, conjunctival pallor, MUAC <20 cm, dyspnea, oral hairy leukoplakia (OHL), oral candidiasis, and gingivitis were significantly associated with <350 CD4 cells/mm^3^. A scoring system based on these variables had a negative predictive value of 87% for excluding subjects with CD4 cell counts <100 cells/mm^3^; however, the positive predictive value for identifying such individuals was low (47%).

**Conclusions:**

Clinical variables correlate with CD4 cell strata in HIV-positive patients with TB. The clinical scoring system had adequate negative predictive value for excluding severe immunosuppression. Clinical scoring systems could be of use to categorize TB/HIV co-infected patients with regard to the timing of ART initiation in settings with limited access to laboratory facilities.

Tuberculosis (TB) is the leading opportunistic infection (OI) globally in people living with HIV and is estimated to be responsible for 400,000 deaths per year in HIV-positive persons (1). In contrast to most other OIs, TB can occur over the whole spectrum of immunodeficiency, although the prevalence increases dramatically with decreasing CD4 cell count (2). Recently, several studies have demonstrated significant survival benefits in HIV-positive subjects initiating antiretroviral therapy (ART) before the completion of anti-tuberculosis treatment (ATT) (3–5). Consequently, the WHO has revised its guidelines, and currently ART is recommended for all TB/HIV co-infected individuals, irrespective of CD4 cell counts (6).

Importantly, the benefit of starting ART early during the intensive phase of ATT is related to CD4 cell levels; a reduction in mortality with ‘immediate ART’ (initiated within the first 2 weeks of ATT) has been shown for patients with severe immunosuppression, but not for those with less advanced HIV disease (3–5, 7). Hence, current WHO guidelines recommend ART initiation within 2 weeks after starting ATT in patients with CD4 cell count<50 cells/mm^3^, and within 8 weeks for subjects with less advanced immunosuppression (6). Although early initiation of ART during ATT reduces the risk of death among severely immunosuppressed persons, this strategy may also lead to more complications during treatment, such as an increased incidence of immune reconstitution inflammatory syndrome (IRIS) (8–10) and a higher risk of severe adverse events in patients with tuberculous meningitis (11).

Assessment of immunosuppression in HIV-positive patients is thus still necessary to determine the timing of ART initiation. This relies on the measurement of CD4 cell levels, in combination with the WHO clinical staging system. However, the performance of the WHO staging system for identification of subjects eligible to initiate ART is suboptimal (12). In particular, the WHO staging system cannot be used to categorize patients with concomitant TB with regard to the degree of immunosuppression, since TB is included as a staging variable. Consequently, CD4 cell measurement is the sole reliable method for the assessment of immunosuppression among subjects with TB/HIV co-infection.

Although CD4 cell technology has been introduced in many locations along with scaling up of ART, this method remains unavailable for many persons living with HIV and may not be cost-effective in low-income countries (13). In order to achieve greater ART coverage in resource-limited settings, greater proportions of patients will receive both ATT and ART within the primary health care system. The need for CD4 cell testing constitutes an important obstacle for efficient decentralization of HIV care to peripheral health levels. Therefore, alternative robust methods that may be used for the assessment of immunosuppression and that do not require laboratory facilities would facilitate management of HIV-infected individuals with TB.

We hypothesized that clinical parameters associated with HIV-related immunosuppression correlate with low CD4 cell counts in HIV-positive patients with TB. Such correlations would allow for the construction of a clinically based scoring system that could be used to categorize patients according to the degree of immunosuppression, and could help to identify subjects who need to start ART early during the course of ATT. Since the clinical manifestations of HIV/AIDS and TB are similar, we also determined the distribution of clinical variables in HIV-negative patients with TB, in order to identify parameters primarily related to HIV infection.

## Patients and methods

### Patients

Individuals diagnosed with TB were consecutively screened for eligibility in TB outpatient clinics at six health centers, one zonal hospital (Bishoftu), and one regional hospital (Adama) in the Oromia Regional State of Ethiopia, between September 2010 and March 2012. The following inclusion criteria were applied: 18 years or older, TB diagnosis according to Ethiopian National Guidelines (14), and consent to HIV testing. Patients with previous or current ART, ATT for more than 2 weeks for a current episode of TB, or those who had received ATT within the preceding 6 months were excluded, as well as subjects not residing in the catchment area of the respective clinics.

Patients in whom acid-fast bacilli were detected in sputum were classified as smear-positive cases. The diagnosis of smear-negative pulmonary TB was established in patients with symptoms compatible with TB, who had repeatedly negative sputum smear microscopy, no response to broad-spectrum antibiotic therapy, and chest X-ray lesions suggestive of TB. For a diagnosis of peripheral lymph node TB, a compatible fine-needle aspirate cytology result was required. Other forms of extrapulmonary TB were diagnosed using targeted investigations, depending on disease manifestation.

HIV serostatus was determined using rapid tests, according to Ethiopian National Guidelines (15). Positive results by KHB (Kehua Bio-engineering Co, Shanghai, China) were immediately followed by testing with Statpack (HIV 1/2 Stat-Pak, Chembio Diagnostic Systems Inc., 3661 Horseblock Rd, Medford, New York, USA) for confirmation of HIV infection. Further testing was performed by Unigold (Uni-Gold TM HIV, Trinity Biotech Plc., Bray, Co. Wicklow, Ireland) when discordant results were obtained with these two assays. Patients diagnosed with HIV infection were referred to HIV clinics in the same health facility for further management and were considered for ART initiation in accordance with Ethiopian national guidelines (16). These guidelines recommend starting ART either after 2–8 weeks or after completion of the intensive phase of ATT, depending on CD4 levels or clinical disease severity, in case CD4 cell counts are not possible to obtain.

### Methods

TB clinic nurses, who received detailed and repeated training by the research group members on the study protocol, performed all study investigations. These nurses had two different levels of qualification (diploma in nursing and bachelor of science). All nurses involved in the study had received training required for being employed in a public Ethiopian TB clinic. A full-day training session with all staff from the participating clinics was arranged by the study team every 6 months for the duration of the study. These training sessions encompassed details of the structured questionnaire and instructions on how to perform patient interviews and physical examination, with particular focus on recognition of various symptoms and clinical findings. The investigators performed once weekly monitoring of the study procedures throughout its duration. This included crosschecking of all relevant data using the clinic registers and records as data sources. In association with these visits, training with each study investigator was repeated. Data clerks entered the data recorded on the questionnaires into a database continuously, with repeated crosschecking of all entries.

A structured questionnaire was used for the collection of study data. This questionnaire focused on disease history and symptoms that were considered to be common or typical of HIV-related immunosuppression, such as bedridden state, hospitalization, cough, dyspnea, fever, weight loss, anorexia, lymph node enlargement, skin rash, diarrhea, and odynophagia.

The degree of wasting was estimated both by calculating the body mass index (BMI; based on measurement of height and weight at the time of inclusion) and by measurement of mid-upper arm circumference (MUAC; using a designated measuring tape to the nearest 0.5 cm). The physical examination included the following items: conjunctival pallor, oral candidiasis, oral hairy leukoplakia (OHL), gingivitis, cervical lymphadenopathy, skin rash (without further specification), and herpes zoster scar.

Blood was drawn into EDTA tubes for analysis of CD4 cell and hemoglobin levels in conjunction with this visit. These blood samples were transported to one of two laboratories (Adama Regional Laboratory or Bishoftu Zonal Hospital Laboratory). CD4 cell count was determined within 24 hours using flow cytometry according to the instructions of the manufacturer (FACSCount or FACSCalibur, BD Biosciences, respectively). Continuous internal and external quality assurance monitoring was done in both laboratories every 3 months.

Results of these blood tests were released to the study investigators within 1 week after patient inclusion and collection of clinical data; consequently, study staff were blinded to CD4 cell counts.

### Statistical analysis

The study was conducted using a cross-sectional design. Participants were first grouped according to HIV serostatus. HIV-positive patients were then further divided into subgroups based on different CD4 cell count levels used for deciding when to initiate ART (<50, <100, <200, and <350 cells/mm^3^). Continuous variables were categorized based on the distribution in the study population; for BMI and MUAC, the material was separated into quartiles whereas age was managed as a bivariate variable.

Following an initial logistic regression univariate analysis, variables with *p*-values <0.3 were entered into a stepwise logistic regression multivariate analysis. Variables found to be significant (*p*-value of <0.05) for each separate CD4 cell stratum in the respective final regression models were used as items in the final scoring system. The beta coefficients (i.e. the log-transformed odds ratios) from these models were added together to assign a weighted scoring point. The final scores were rounded to the nearest 0.5 integer. In the final model, MUAC was included as a binary variable based on the distribution from the previous step.

The resulting score consisted of variables that had associations with CD4 cell count of <350 cells/mm^3^; Sensitivity, specificity, positive predictive value (PPV), and negative predictive value (NPV) were calculated for patients with CD4 cell counts below 100 cells/mm^3^. Only patients with complete data and no missing values were used in this analysis. Receiver operator characteristic (ROC) analysis was performed and area under the curve (AUC) was calculated for the score at the selected CD4 cell cutoff levels. All analyses were done using IBM SPSS statistics version 20.

## Ethical considerations

Patients provided written informed consent of their confidential participation in the study. For illiterate subjects, an independent witness certified that they had received information about the study and their acceptance of inclusion. The study received ethical approval by the National Ethics Review Committee at the Ministry of Science and Technology of Ethiopia and by the Ethical Committee at Lund University, Sweden.

## Results

### Patient characteristics

**Fig. 1:**
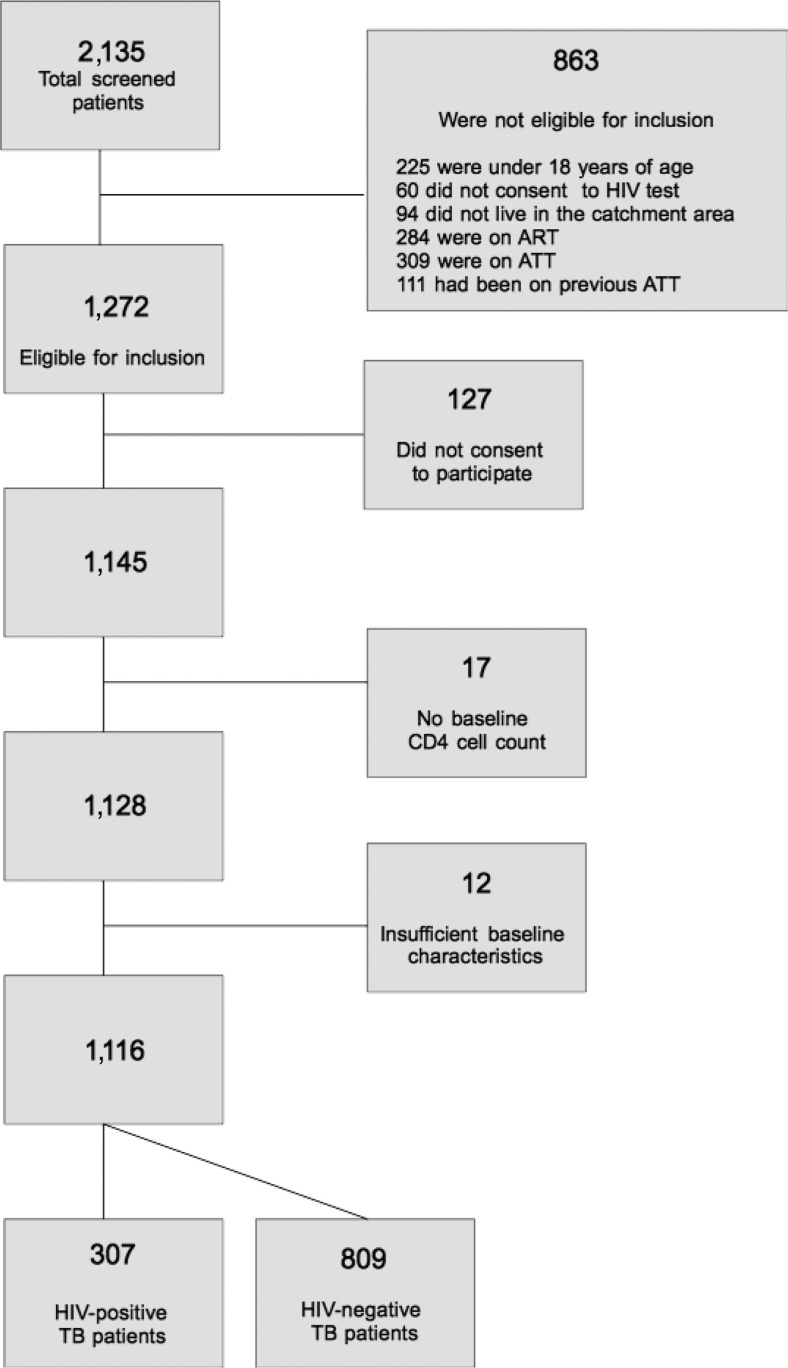
Flow diagram of study participants.

During the inclusion period, 2,135 patients were registered in the study clinics. Among these, 1,116 were included, 307 (27.5%) of whom were HIV-positive. A flow chart of eligible subjects and participants is presented in Fig. 1. Characteristics with regard to HIV serostatus are shown in Table 1. Patients co-infected with HIV were slightly older than HIV-negative subjects (median age 32 vs. 29 years, respectively) but gender distribution was similar in both groups. Most patients reported urban residence. Approximately two thirds of the TB cases were pulmonary, with a higher proportion of smear-negative disease in HIV-positive subjects. Peripheral lymphadenitis was the most frequent cause of extrapulmonary TB, both for HIV-positive and HIV-negative persons. However, no significant differences between HIV-positive and negative subjects were found with regard to type of TB manifestation.

**Table 1:** Background characteristics and comparison between 307 HIV-positive- and 809 HIV-negative-TB patients

		HIV-positive TB-patients (*n*=307)	HIV-negative TB-patients (*n*=809)	OR (95% CI)*
Background data	Median age (years; range)	32 (18–70)	29 (18–80)	–
	Male gender	156 (50.8)	432 (53.4)	–
	Urban residence	274/306 (89.5)	669/807 (82.9)	–
	Rural residence	32/306 (10.5)	138/807 (17.1)	–
	Smear-positive PTB	96 (31.3)	309 (38.2)	–
	Smear negative PTB	96 (31.3)	214 (26.5)	–
	Peripheral lymphadenitis	91 (29.6)	205 (25.3)	–
	Other location of TB	34 (11.0)	91 (11.3)	–
	Median CD4 cell count (IQR)	173 (95–336)	671 (500–883)	–
	Median CD4/CD45% (IQR)	12 (8–18) (*n*=209)	37 (31–43) (*n*=518)	–
Symptoms associated	Oral candidiasis	92 (30.0)	11 (1.4)	5.9 (3.5–9.9)
with HIV diagnosis	Herpes zoster	24/306 (7.8)	4 (0.5)	4.3 (1.6–11.2)
	Skin rash	38 (12.4)	4 (0.5)	2.7 (1.2–5.9)
	Diarrhea	66/305 (21.6)	34/808 (4.2)	1.9 (1.1–3.1)
	MUAC ≥22 cm	105 (30)	409 (50.6)	REF
	MUAC 20–22 cm	92 (30)	239 (29.5)	1.5 (1.0–2.1)
	MUAC 19–20 cm	42 (13.7)	71 (8.8)	2.2 (1.4–3.6)
	MUAC <19 cm	68 (22.1)	90 (11.1)	2.5 (1.6–3.9)
	Odynophagia	117 (38.1)	102 (12.6)	1.6 (1.1–2.4)
	Conjunctival pallor	118 (38.4)	93/806 (11.5)	1.6 (1.1–2.4)
	Previous history of TB	7 (2.3)	40 (4.9)	0.3 (0.1–0.7)

Presented as *n* (%) unless otherwise stated. CD4 cell values in cells/mm^3^.

No significant associations (95% CI) were found for age, gender residence, and type of TB.

* Multivariate associations between HIV diagnosis and the variables.

### Clinical variables associated with HIV co-infection

The following clinical variables were found to be significantly associated with HIV infection in multivariate analysis: oral candidiasis, herpes zoster scar, skin rash, MUAC <20 cm, diarrhea, odynophagia, and conjunctival pallor (in order of decreasing degree of association; Table 1). The median CD4 cell count for HIV + /TB patients was 173 cells/mm^3^ and 671 cells/mm^3^ for HIV-/TB patients (*p*<0.001). Previous TB was less commonly reported by HIV-positive patients.

**Table 2:** Frequency of symptoms and signs in 307 TB patients co-infected with HIV

		Total, *n*=307	CD4<100, *n*=79	CD4 100–350, *n*=155	CD4>350, *n*=73	P*
Background information	Male gender	156 (50.5)	51 (64.6)	71 (45.8)	34 (46.6)	0.022
	Previous TB	7 (2.3)	1 (1.3)	3 (1.9)	3 (4.1)	0.714
Diagnosis of TB	Smear positive PTB	99 (32.2)	29 (36.7)	46 (29.7)	24 (32.9)	0.621
	Smear negative PTB	94 (30.6)	27 (34.2)	46 (29.7)	21 (28.8)	0.422
	Lymphnode TB	87 (28.3)	20 (25.3)	44 (28.4)	23 (31.5)	0.326
	Other location of TB	36 (11.7)	8 (10.1)	22 (14.2)	6 (8.2)	0.760
Symptoms	Bedridden state	81 (26.4)	31 (39.2)	32 (20.6)	18 (24.7)	0.018
	Blood-stained sputum	47/305 (15.4)	15/78 (19.2)	20/154 (13.0)	12 (16.4)	0.906
	Cough	190/306 (62.1)	55/78 (70.5)	100 (64.5)	35 (47.9)	0.001
	Diarrhea	66/305 (21.6)	22/78 (28.2)	32/154 (20.8)	12 (16.4)	0.048
	Diarrhea, recurrent	43/302 (14.2)	16/77 (20.8)	21/153 (13.7)	6/72 (8.3)	0.015
	Fever	253 (82.4)	68 (86.1)	128 (82.6)	57 (78.1)	0.063
	Hospitalized	30/305 (9.8)	14 (17.7)	10/153 (6.5)	6 (8.2)	0.005
	Loss of appetite	249 (81.1)	71 (89.9)	127 (81.9)	51 (69.9)	0.001
	Night sweats	256 (83.4)	70 (88.6)	126 (81.3)	60 (82.2)	0.043
	Odynophagia	117 (38.1)	35 (44.3)	56 (36.1)	26 (35.6)	0.173
	Shortness of breath	148/305 (48.5)	46/78 (59)	82/154 (53.2)	20 (27.4)	0.000
	Significant weight loss	254/306 (83.0)	67 (84.8)	131/154 (85.1)	56 (76.7)	0.150
Clinical findings	Cervical lymph node enlargement	58/306 (19.0)	9/78 (11.5)	32 (20.6)	17 (23.3)	0.015
	Conjunctival pallor	118 (38.4)	44 (55.7)	53 (34.2)	21 (28.8)	0.004
	Gingivitis	24/305 (7.9)	9/78 (11.5)	13/154 (8.4)	2 (2.7)	0.010
	Herpes zoster	24/306 (7.8)	6 (7.6)	14/154 (9.1)	4 (5.5)	0.685
	Oral candidiasis	92 (30.0)	32 (40.5)	45 (29)	15 (20.5)	0.003
	Oral hairy leukoplakia	15/306 (4.9)	9 (11.4)	4/154 (2.6)	2 (2.7)	0.005
	Skin rash	38 (12.4)	10 (12.7)	19 (12.3)	9 (12.3)	0.669
Grouped continuous	BMI					0.001
variables	BMI ≥19.5	73 (23.8)	12 (15.2)	39 (25.2)	22 (30.1)	
	BMI 17.5–19.49	82 (26.7)	23 (29.1)	39 (25.2)	20 (27.4)	
	BMI 16–17.49	83 (27.0)	19 (24.1)	47 (30.3)	17 (23.3)	
	BMI <16	69 (22.5)	25 (31.6)	30 (19.4)	14 (19.2)	
	MUAC					<0.001
	MUAC ≥22	105 (34.2)	15 (19)	57 (36.8)	33 (45.2)	
	MUAC 20–22	92 (30.0)	19 (24.1)	48 (31.0)	25 (34.2)	
	MUAC 19–20	42 (13.7)	17 (21.5)	19 (12.3)	6 (8.2)	
	MUAC <19	68 (22.1)	28 (35.4)	31 (20.0)	9 (12.3)	
	Age (≥33)	153 (49.8)	47 (59.5)	75 (78.4)	31 (42.5)	0.031

Presented as *n* (%) unless otherwise stated. CD4 cell values in cells/mm^3^.

* Correlation of variable to CD4. Binary variables tested with Mann–Whitney U test, Continuous variables tested with Spearman's rank correlation coefficient.

### Correlation of clinical variables to CD4 cell count strata in HIV-positive patients

The frequencies of various clinical variables were determined for different CD4 cell strata (Table 2). The degree of wasting (estimated either by BMI and MUAC) was associated with more advanced immunosuppression (CD4 cell count <100 cells/mm^3^, *p*<0.001). As BMI and MUAC showed colinearity, MUAC, which showed the stronger correlation of the two, was included in the final model as a binary variable based on the distribution from the previous step. However, reported history of weight loss showed no such correlation (*p*=0.15). In multivariate regression analysis, male gender, age>32 years, conjunctival pallor, shortness of breath, OHL, oral candidiasis, and MUAC <20 cm were independently associated with CD4 cell counts <350 cells/mm^3^ (Table 3).CD4 cell counts <50 cells/mm^3^ were associated with OHL and MUAC <20 cm.

**Table 3:** Significant variables in multivariable analysis

	CD4 cell count <50		CD4 cell count <100		CD4 cell count <200		CD4 cell count <350			
						
	β	Adjusted OR (95% CI)	β	Adjusted OR (95% CI)	β	Adjusted OR (95% CI)	β	Adjusted OR (95% CI)	Totalβ	Rounded score
Male gender			0.8	2.3 (1.3–4.2)					0.8	1
Age≥33 years					0.5	1.7 (1.0–2.8)			0.5	0.5
Conjunctival pallor			0.8	2.4 (1.4–4.3)					0.8	1
Shortness of breath					0.7	2.1 (1.3–3.4)	1.0	2.9 (1.6–5.3)	1.7	1.5
OHL	2.4	7.5 (2.2–25.2)							2.4	2.5
Oral candidiasis					0.6	2.1 (1.2–3.7)			0.6	0.5
Gingivitis					1.4	4.3 (1.2–15.8)			1.4	1.5
MUAC <20 cm	1.3	4.6 (2.0–10.5)	1.0	2.8 (1.6–4.9)	0.7	2.2 (1.3–3.6)	0.9	2.4 (1.2–4.5)	3.9	4
AUC	0.72		0.72		0.71		0.68		0.72	

Presented according to CD4 cell count strata.

### A clinical scoring system for immunosuppression based on clinical variables

Based on the multivariate analysis, a weighted clinical score was developed by the addition of the beta coefficients of variables with significant independent associations with CD4 cell strata (Table 3). The maximum score that could be obtained with this system was 12.5 points. The clinical variables were assigned the following scoring points: MUAC <20 cm 4, OHL 2.5, gingivitis 1.5, shortness of breath 1.5, conjunctival pallor 1, male gender 1, age>32 years 0.5, oral candidiasis 0.5 points. Among these variables, MUAC <20 cm showed the strongest correlation with CD4 cell strata, whereas OHL, despite being a relatively rare finding, was associated with the lowest CD4 cell strata, and thus rendered a high score value.

**Table 4:** Distribution of scoring points among 302* HIV-positive patients with TB, and performance for identification of patients with <100 CD4 cells/mm^3^

Score	CD4 < 100 (n)	CD4 100–350 (n)	CD4 > 350 (n)	Sensitivity (%)	Specificity (%)	Positive predictive value (PPV) (%)	Negative predictive value (NPV) (%)
0	1	10	9	100	8	25	95
1	3	17	18	99	24	27	93
2	11	31	15	95	44	30	87
3	10	26	11	81	61	33	85
4	5	17	4	68	70	37	84
5	3	12	5	61	78	41	84
6	11	15	6	57	87	47	82
7	15	10	4	43	93	53	78
>7	18	14	1	23	100	55	75
Total	77	152	73				

CD4 cell values in cells/mm^3^.

Score cutoff rounded up to nearest integer. Sensitivity and PPV of being at threshold or above. Specificity and negative predictive value of being at threshold or below.

* Only patients with no missing values were included in this analysis.

### Performance of the clinical scoring system

The distribution of the clinical scoring points for the study population is shown in Table 4. The overall AUC of the model for identification of patients with CD4 cell counts <100 cells/mm^3^ was 0.721. The resulting scoring system could exclude most patients without advanced immunosuppression (CD4 cell counts below 100 cells/mm^3^); 101/116 (NPV 87%) patients with scores ≤2 had CD4 cell counts above this threshold level. There was a clear trend for lower CD4 levels in patients with higher scores, however the PPV remained low even at higher points (47% for a score of 6 points or more) for identification of patients with CD4 <100 cells/mm^3^.

## Discussion

In this population of HIV-positive adults with TB consecutively recruited in Ethiopian out-patient clinics, several clinical variables were found to correlate with CD4 cell strata used to decide the timing for initiating ART during ATT. Based on these findings, a clinical scoring system was developed for the categorization of patients with regard to these CD4 cell strata.

Determining the degree of immunosuppression in HIV infection relies on the measurement of CD4 cell levels in peripheral blood, which is well recognized to have a strong predictive value for HIV disease progression (17). As a consequence, CD4 cell counts are used in all guidelines for recommendations on when to initiate ART. The need for CD4 cell testing constitutes an important obstacle for further roll-out of ART, especially at primary health care level in low-income countries. In such facilities, regular and reliable access to laboratory facilities may not be possible to achieve. Therefore, alternative robust methods for assessing the degree of immunosuppression are needed; a scoring system completely based on clinical data would be the most attractive option. In current guidelines, the WHO staging system is recommended in settings without access to CD4 cell testing. However, validation data for this system for the purpose of ART initiation are scarce (18, 19). The fact that TB in itself is used as a WHO staging criterion hampers its use in TB/HIV co-infected patients. Furthermore, the timing of ART during the intensive phase of ATT in settings without access to CD4 cell testing has not been the subject of specific investigation, and the WHO guidelines do not specify how to manage patients in this situation.

Scoring systems are used in various fields of medicine for determining the likelihood of certain diagnoses (e.g. pulmonary embolism (20), but also for classification of disease severity (e.g. NYHA scoring system for heart failure (21). An advantage of scoring systems as compared to screening algorithms is that they allow for a two way identification of thresholds (both identification of severe disease and exclusion of disease), which make such systems especially suitable for prediction of continuous variables like the degree of immunosuppression. Furthermore, a clinically based scoring system may be repeated during follow up of individual patients, which might allow for identification of subjects with disease progression.

Scoring systems could potentially be of great use in decentralized HIV care. For example, Lynen et al. have presented a clinically based scoring system for estimating the likelihood of virological treatment failure in Cambodian patients receiving ART, intended for use in settings without access to viral load testing (22). To our knowledge, clinical correlates of CD4 cell counts in HIV/TB co-infection has hitherto only been investigated in a small hospital-based study of patients with concomitant TB in South Africa (23). These authors found that CD4 cell counts <200 cells/mm^3^ were associated with a BMI <18 kg/m^2^, low Karnofsky score and low hemoglobin concentration.

Since no scoring system for the assessment of immunosuppression in HIV/TB patients has previously been proposed, we aimed to determine the feasibility of our hypothesis by construction of an optimized system. We used CD4 cell count to categorize subjects with regard to the degree of immunosuppression. Our study thus investigates the concurrent validity of the scoring system to predict CD4 cell strata; we did not aim to explore the potential predictive capacity of the system for outcomes such as incident opportunistic diseases or mortality.

We used logistic regression to identify variables with correlation to CD4 cell strata, and used those showing significant associations as items in the scoring system. Other statistical methods could be considered for this purpose, for instance an algorithmic approach (24); however, we think that such a strategy would have been too complex for this exploratory study. In order to find the best possible algorithm based on our data, we decided to weight variables significantly associated with CD4 cell count strata using beta coefficients. Beta coefficients have been used by other researchers for the purpose of assigning variables a relative weight (25–28). Although the value of weighting variables for the development of scoring systems has been debated (29, 30), Streiner et al. found a benefit of weighting in analyses containing <40 items, which is the case for this study (31).

Our scoring system is based on variables that can be collected by health professionals with limited training. Considering that it is based on clinical parameters, this scoring system has an adequate NPV for CD4 cell counts ≥100 cells/mm^3^ for subjects with scores of 2 or less. This would correctly classify 87% of persons with less advanced immunosuppression, for whom ART initiation after 2 weeks of ATT (‘immediate ART’) is not indicated. Although there was a clear trend of higher scores with decreasing CD4 cell counts, the PPV was below 50%, making it unsuitable for direct identification of patients with advanced immunosuppression. Yet, subjects with a high score (6 or greater) were unlikely to have CD4 cell counts above 350 cells/mm^3^ (11 out of 95 patients, 12%).

This scoring instrument might thus be used for identification of subjects with less advanced immunosuppression (CD4 ≥100 cells/mm^3^). According to current guidelines, such persons are eligible to start ART during the intensive phase of ATT, but do not qualify for immediate ART. Thus, categorization based on our scoring system would permit a longer time interval between the initiation of these two treatments for such patients. This strategy may be of benefit in order to assess the tolerability of ATT and treatment adherence before ART initiation. A CD4 cell count <100 cells/mm^3^ was used to define severe immunosuppression. This range is slightly broader than that used to identify subjects eligible for immediate ART in the current WHO guidelines (50 cells/mm^3^). Indeed, there is no exact threshold for reduced mortality with immediate ART; for instance, Blanc et al. demonstrated increased survival with this strategy for patients with CD4 cell counts <200 cells/mm^3^ (5). Among our patients, 38% (115/302) had scores of 2 or less, showing that more than one third of co-infected patients might be classified in this way.

However, 15 out of 115 subjects (13%) with CD4 cell counts <100 cells/mm^3^ would not be recognized as candidates for ‘immediate ART’. Whether this would confer an increased risk of death among such individuals cannot be determined from our study design. Since current guidelines recommend care-providers to consider starting ART during the first 2 months of ATT for all TB patients, this misclassification would probably not lead to great delays in ART initiation. Furthermore, since our clinical scoring system also correlates with markers known to be associated with poor outcome among TB patients, the absence of such manifestations suggest that this subgroup of patients might have a lower risk of mortality (despite low CD4 cell counts) (32).

Although early initiation of ART during ATT leads to decreased overall mortality in patients with advanced immunosuppression, this approach may also have certain negative consequences. The risk of IRIS is clearly increased with early initiation of ART (8), and the outcome of TB-associated IRIS in patients managed in health centers is not well known. Furthermore, the incidence of severe adverse events is increased in patients with tuberculous meningitis (11), which is common in HIV-infected persons (33). Correct identification of this condition is difficult even in high-resource settings. In our material only one TB patient was diagnosed with meningitis. There is a definite need for targeted studies on TB in HIV-positive patients with advanced immunosuppression receiving care in health centers, especially with regard to the risk of adverse events and IRIS.

The strongest association with low CD4 cell counts was found for MUAC <20 cm. By itself, this parameter had an AUC of 0.62 for identification of patients with CD4 cell counts <100 cells/mm^3^. BMI showed intercorrelation with MUAC, but MUAC proved to be a stronger predictor for low CD4 cell counts. MUAC is a reliable and easy way of measuring the degree of wasting and can also be performed in bedridden patients (32), and has furthermore been shown to be highly predictive of mortality in persons with TB. It is possible that these parameters for wasting would show different correlations to CD4 cell counts in other settings and populations.

In order to determine the contribution of active TB to the distribution of clinical variables among co-infected subjects, we included HIV-negative TB patients from the same uptake area. We observed significant differences in clinical presentation with regard to HIV serostatus. Clinical parameters associated with HIV infection were typical features of HIV-related immunosuppression such as oral candidiasis, herpes zoster, gingivitis and diarrhea, low MUAC, odynophagia and conjunctival pallor, most of which are part of the WHO staging system. Interestingly, several WHO staging variables did not correlate to CD4 cell count strata (e.g. weight loss, fever, diarrhea). This supports previous reports showing a low performance for identification of subjects with low CD4 cell levels using the WHO staging system (34).

To our knowledge, this study is the largest investigation of associations between clinical variables and degrees of immunosuppression in TB/HIV co-infected subjects. Since most participants were recruited in health centers the study population is representative of TB/HIV co-infected patients in Ethiopia. The collection of data was performed by regular clinic staff with limited medical training, which might have decreased the accuracy of detected symptoms and signs. However, this also shows the feasibility of the clinical scoring approach in routine practice. The risk of bias in data collection with regard to CD4 cell levels was eliminated since these results were released after completion of the scoring examination.

### Limitations

This proof-of-concept study has several limitations. In order to test our main hypothesis – the feasibility of constructing a clinical scoring system to assess immunosuppression in HIV/TB co-infected subjects – we chose a model optimized for the study population. This might have reduced the validity of the scoring system in other settings; testing of the scoring system in other geographical areas is necessary. In addition, assessment of the predictive validity and interobserver reliability is required before implementation in clinical practice can be considered.

We chose to define threshold levels for MUAC based on the distribution of values in our study population instead of using standard levels. Although this provided more relevant data for our purpose, these levels may have to be revised and modified for use in other settings. Furthermore, we included age and gender in our scoring system. These parameters could differ between populations. Different reasons may exist for the associations between these variables and CD4 cell strata. Previous studies have shown variations in CD4 cell levels with regard to age and gender (35), suggesting that inclusion of these variables is justified. We also constructed an algorithm excluding age and gender (data not shown); this model had a slightly worse performance (AUC 0.69 vs. 0.72).

For the diagnosis of TB, Ethiopian national guidelines were used, and microbiological confirmation was not required for inclusion. Although some patients may have been incorrectly diagnosed with TB, this reflects the normal situation in sub-Saharan Africa, where access to microbiological diagnostic methods for TB remains restricted.

## Conclusions

Several clinical variables that can be recorded by primary health care professionals with limited training were associated with the degree of immunosuppression among Ethiopian patients with TB and HIV co-infection. A scoring system based on these parameters for categorization of patients with regard to the optimal time point for ART initiation during ATT in settings with restricted access to laboratory facilities is feasible; however, further development and validation of this algorithm is needed.
